# Concurrent YAP/TAZ and SMAD signaling mediate vocal fold fibrosis

**DOI:** 10.1038/s41598-021-92871-z

**Published:** 2021-06-29

**Authors:** Ryosuke Nakamura, Nao Hiwatashi, Renjie Bing, Carina P. Doyle, Ryan C. Branski

**Affiliations:** 1 Department of Rehabilitation Medicine, NYU Grossman School of Medicine, New York, NY USA; 2 Department of Otolaryngology-Head and Neck Surgery, Graduate School of Medicine, Kyoto University, Kyoto, Japan; 3 Department of Otolaryngology-Head and Neck Surgery, NYU Grossman School of Medicine, 240 East 38th Street, Suite 1774, New York, NY 10016 USA

**Keywords:** Translational research, Molecular medicine

## Abstract

Vocal fold (VF) fibrosis is a major cause of intractable voice-related disability and reduced quality of life. Excision of fibrotic regions is suboptimal and associated with scar recurrence and/or further iatrogenic damage. Non-surgical interventions are limited, putatively related to limited insight regarding biochemical events underlying fibrosis, and downstream, the lack of therapeutic targets. YAP/TAZ integrates diverse cell signaling events and interacts with signaling pathways related to fibrosis, including the TGF-β/SMAD pathway. We investigated the expression of YAP/TAZ following vocal fold injury in vivo as well as the effects of TGF-β1 on YAP/TAZ activity in human vocal fold fibroblasts, fibroblast-myofibroblast transition, and TGF-β/SMAD signaling. Iatrogenic injury increased nuclear localization of YAP and TAZ in fibrotic rat vocal folds. In vitro, TGF-β1 activated YAP and TAZ in human VF fibroblasts, and inhibition of YAP/TAZ reversed TGF-β1-stimulated fibroplastic gene upregulation. Additionally, TGF-β1 induced localization of YAP and TAZ in close proximity to SMAD2/3, and nuclear accumulation of SMAD2/3 was inhibited by a YAP/TAZ inhibitor. Collectively, YAP and TAZ were synergistically activated with the TGF-β/SMAD pathway, and likely essential for the fibroplastic phenotypic shift in VF fibroblasts. Based on these data, YAP/TAZ may evolve as an attractive therapeutic target for VF fibrosis.

## Introduction

Vocal fold (VF) fibrosis is a major cause of intractable dysphonia commonly resulting in reduced quality of life as well as occupational and social limitations and profound healthcare expesnses^1–4^. The etiology of VF fibrosis is diverse; however, from a mechanistic perspective, activation of fibroblasts to the more metabolically active myofibroblast phenotype indicates a transition from normal to more fibrotic tissue. Myofibroblasts have significantly increased fibroplastic capacity related to both extracellular matrix (ECM) synthesis and contractility^5–8^. Accumulation of fibrous ECM increases tissue stiffness and decreases pliability required for vibratory function. Therapeutic options for vocal fold fibrosis, such as excision of fibrotic regions, injection of biomaterials, and steroid treatments are associated with risk of iatrogenic damage and inconsistent outcomes^9–15^. Novel biomaterials and growth factor therapies may hold clinical promise^16–18^, but standard and efficacious techniques to address this recalcitrant clinical issue remain undescribed.

Transforming growth factor β (TGF-β) is known to drive the phenotypic transition from fibroblasts to the fibrotic myofibroblast^19,20^. TGF-β signaling through its corresponding receptors activates regulatory SMADs (R-SMADs), and in turn, R-SMADs stimulate transcription of fibroplastic genes with co-SMADs. Based on data from our laboratory and others, TGF-β/SMAD signaling is likely an ideal therapeutic target for tissue fibrosis. Our group confirmed that knock down of SMAD3 via siRNA inhibited myofibroblast differentiation in human vocal fold fibroblasts in vitro and preliminary data may suggest some therapeutic effectiveness in vivo^21,22^. However, activation of SMAD signaling does not occur in isolation and likely results in upregulation of R-SMAD inhibitors^23^. This complexity and the potential for such therapies to disrupt endogenous SMAD inhibitory actions pose a significant challenge to therapeutic approaches targeting only SMADs for fibrosis.

The development of novel, targeted therapeutics for vocal fold fibrosis requires further investigation into the complexities of wound repair. The Hippo signaling pathway was originally identified in *Dropsophila melanogaster* to restrict tissue growth^24–28^. More recently, the role of Hippo signaling expanded to include multifaceted roles in cell survival, differentiation, organ development, and tissue regeneration^29,30^. Yes-associated protein (YAP) and transcriptional co-activator with PDZ binding motif (TAZ) have a central role in the Hippo pathway to regulate activity of corresponding transcriptional factors, such as transcriptional enhanced associate domain (TEAD) family molecules. Although the Hippo pathway is modulated by soluble factors, cell–cell junctions, and ECM, these signals influence YAP/TAZ nuclear translocation and its role to support TEAD^31–34^.

In addition, several signaling pathways related to fibrosis, including Wnt and Rho, interact with YAP/TAZ further suggesting Hippo involvement in pathological fibrosis. For example, pharmacological inactivation of YAP prevented carbon tetrachloride-induced hepatic fibrosis^35^. In addition, null mutation in Salvador, a negative regulator of YAP, exacerbated collagen deposition and α-smooth muscle actin (αSMA) expression in murine renal fibrosis^36^. TGF-β/SMAD signaling is also mediated by YAP/TAZ. TGF-β signaling leads to nuclear localization of YAP and/or TAZ and upregulates TEAD target genes^30,37–39^. Mechanistically, YAP/TAZ binds Smad2/3 upon TGF-β stimulation and mediates nuclear translocation in mouse embryonic stem cells, HaCaT human keratinocytes, and human conjunctival fibroblasts^39–41^. YAP and TAZ are differentially activated in distinct cell types and their interaction with SMAD signaling also appears variable.

Data from multiple tissues suggest a putative role for YAP/TAZ in vocal fold fibrosis. However, YAP/TAZ function in vocal fold fibroblasts (VFFbs) has not been described. We sought to localize YAP/TAZ in normal vocal folds and in response to iatrogenic injury. We also investigated the effects of TGF-β1 on YAP/TAZ activity in human vocal fold fibroblasts and the role of YAP/TAZ in fibroblast-myofibroblast transition. We then investigated YAP/TAZ as a regulator of SMAD2/3 nuclear accumulation. Collectively, these data provide additional insight into pathological fibrosis with the ultimate goal of developing novel, targeted therapeutics for this challenging patient cohort.

## Results

### YAP/TAZ increased in response to iatrogenic vocal fold injury

**Figure 1:**
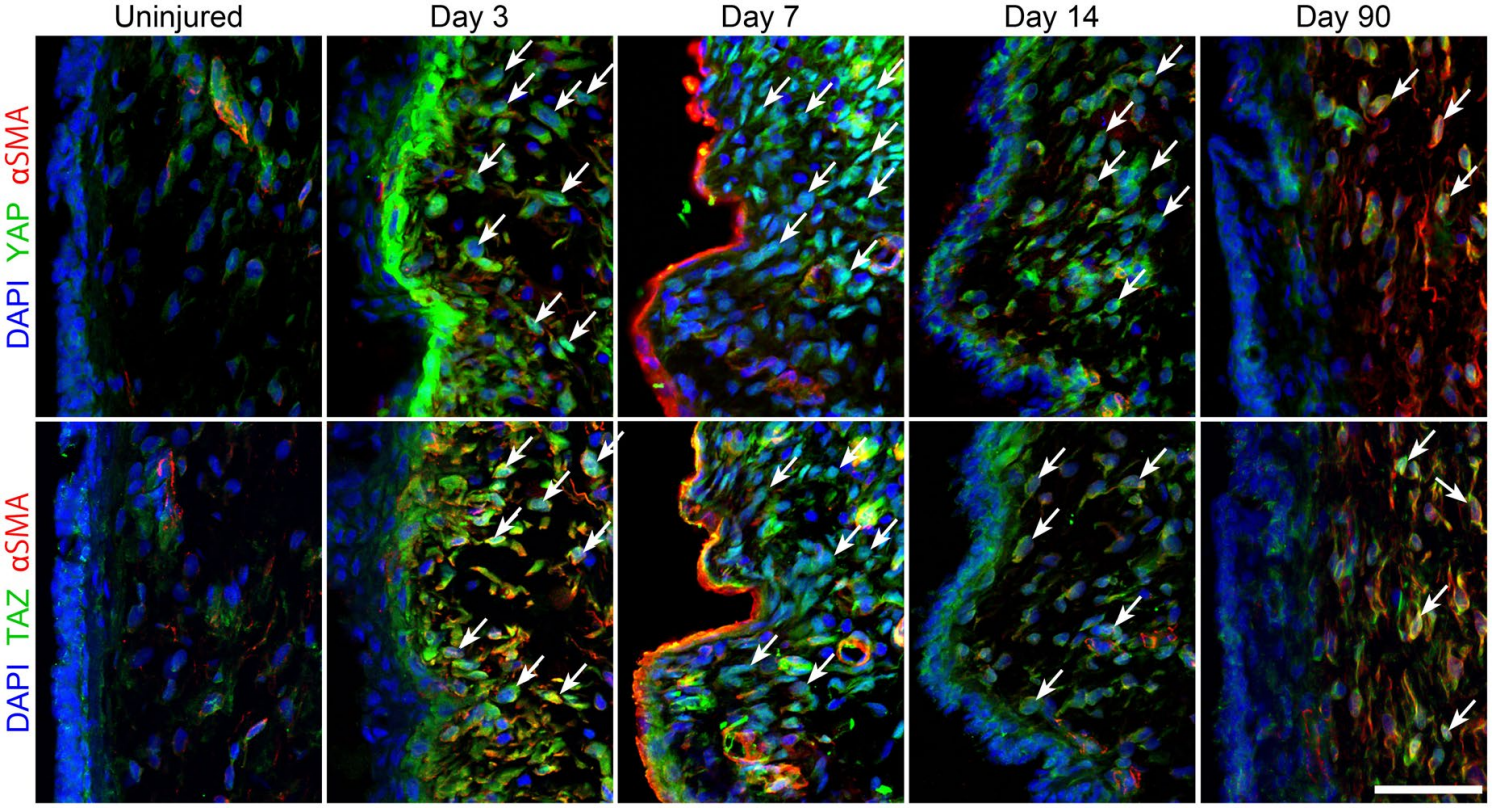
YAP/TAZ distribution in normal and injured rat vocal folds. Tissue was harvested 3, 7, 14, and 90 days following iatrogenic vocal fold injury. Cell nuclei were counterstained using DAPI (bar = 50 µm; arrows indicate nuclear localization of YAP and TAZ, representative images shown).

As an initial step, we immunolocalized YAP/TAZ in rat vocal folds at baseline and in response to injury. In this model, significant fibrosis was reported ~ 2 months after injury^42^. At baseline, faint cytoplasmic YAP and TAZ staining was observed in the lamina propria (Fig. 1) as well as increased intensity in αSMA -positive pericytes located in tubular structures. Following injury, YAP and TAZ staining intensified in both epithelial and mesenchymal cells. Intense staining in mesenchymal cells persisted at both 7 and 14 days after injury; this positivity frequently overlapped with DAPI-stained nuclear regions. The intensity of YAP and TAZ staining decreased at 90 days, but remained elevated compared to uninjured tissue. Staining for YAP and TAZ were observed in nuclear regions at 90 days in some cells.

### TGF-β1 activated YAP/TAZ in human VF fibroblasts

**Figure 2:**
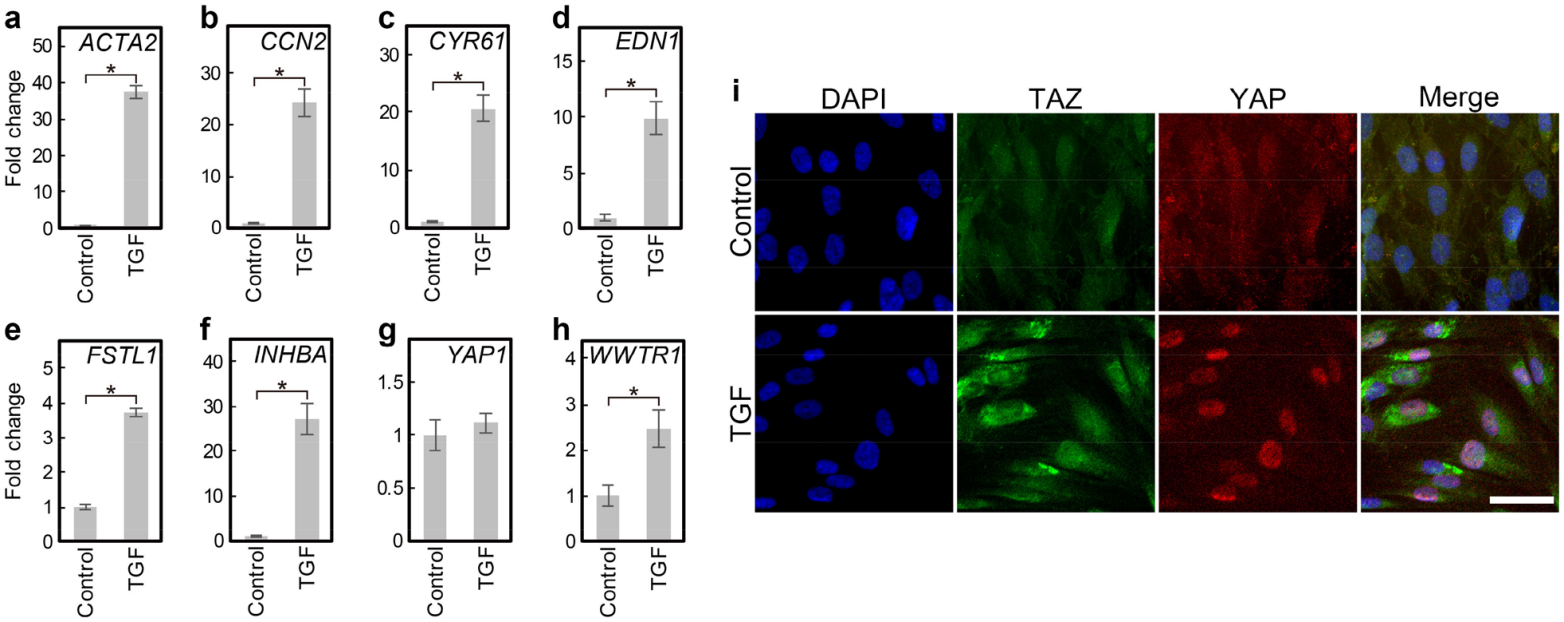
TGF-β1 activated YAP/TAZ in HVOX. HVOX fibroblasts were treated with or without 10 ng/mL TGF-β1 for 24 h. Expression of *ACTA2* (**a**), target genes of YAP/TAZ (**b**, *CCN2*; **c** *CYR61*; **d** *EDN1*; **e** *FSTL1*; **f** *INHBA*), *YAP1* (**g**), and WWTR1 (**h**) were analyzed by qPCR (Data shown as mean ± SD; n = 3; *p < 0.05, Student’s *t*-test). YAP/TAZ nuclear localization was examined by immunostaining (**i**; bar = 50 µm).

Activation of YAP/TAZ was examined in vitro in HVOX fibroblasts, an immortalized human VF fibroblast cell line created by our group and used extensively^43^. TGF-β1, a known mediator of the myofibroblastic phenotype, upregulated *ACTA2* as well as *CCN2*, *CYR61*, *EDN1*, *FSTL1*, and *INHBA*-suggesting YAP/TAZ activation (Fig. 2a–f)^44–48^. YAP mRNA expression was unchanged (Fig. 2g). TAZ (gene name: *WWTR1*) mRNA expression was approximately 2 times higher in TGF-β1-treated cells compared to control (Fig. 2h). TGF-β1 increased YAP/TAZ immunostaining in the nuclei (Fig. 2i), confirming activation of YAP/TAZ. TAZ staining also increased in the cytoplasm, likley reflecting TAZ mRNA upregulation.

### YAP/TAZ inhibition decreased TGF-β1-induced fibrotic gene expression

**Figure 3:**
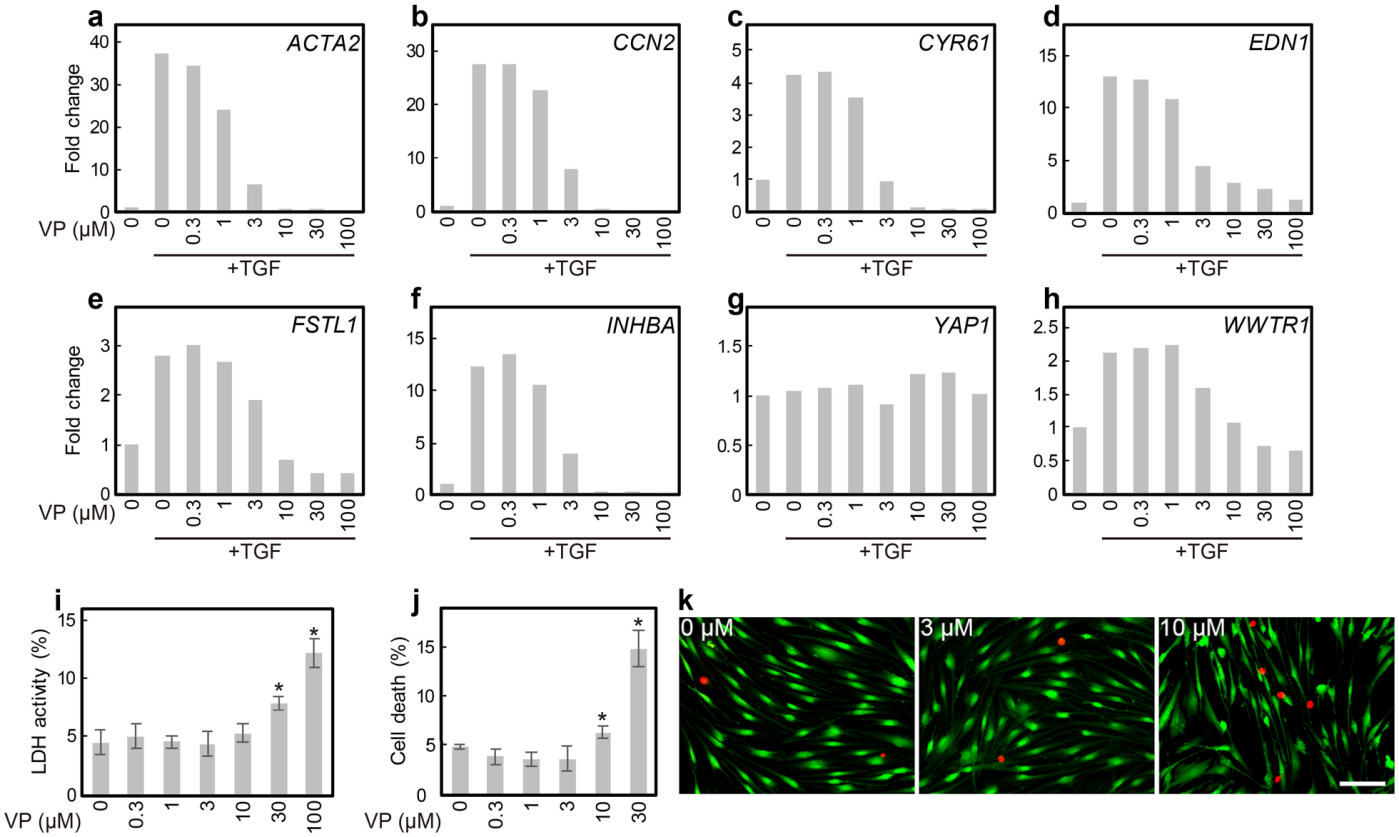
Verteporfin inhibited TGF-β1-induced YAP/TAZ activation in HVOX fibroblasts. HVOX fibroblasts were treated with or without 10 ng/mL TGF-β1 and 0–100 µM verteporfin (VP) for 24 h. Expression of *ACTA2* (**a**), target genes of YAP/TAZ (**b** *CCN2*; **c** *CYR61*: **d** *EDN1*; **e** *FSTL1*; **f** *INHBA*), *YAP1* (**g**), and WWTR1 (**h**) were analyzed by qPCR. Cytotoxicity of VP was analyzed by LDH (**i**) and LIVE/DEAD cell viability assays (j; data shown as mean ± SD in i and j; n = 5; *p < 0.05, Dunnett's test). Representative images of live (green) and dead (red) cells respectively stained with calcein AM and ethidium homodimer-1 (k; bar = 100 µm).

HVOX fibroblasts were treated with verteporfin, a YAP/TAZ inhibitor, to interrogate YAP/TAZ involvement in myofibroblast differentiation. TGF-β1-mediated gene regulation was inhibited by verteporfin in a concentration-dependent manner (Fig. 3a–f). Expression of YAP was not altered by verteporfin (Fig. 3g). In contrast, *WWTR1* upregulation by TGF-β1 was reversed by verteporfin (Fig. 3h). The inhibitory effect of vertporfin was observed at ~ 1 µM, but was most effective between 10 and 30 µM. To confirm this inhibitory activity of verteporfin was not related to cytotoxicity, lactate dehydrogenase (LDH) assay and live/dead-cell count assays were performed. Verteporfin was not associated with significant cytotoxicity at 3 µM (Fig. 3i–k); this concentration inhibited YAP/TAZ-related gene expression and was employed in all subsequent experimentation.

**Figure 4:**
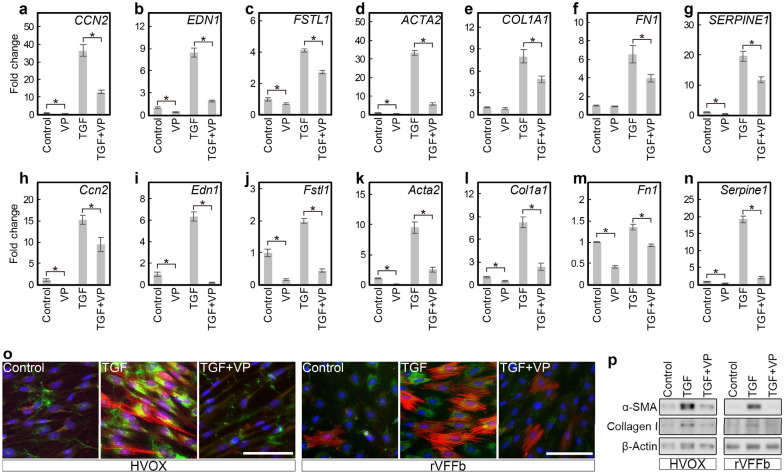
Verteporfin inhibited TGF-β1-induced fibroplastic phenotype changes in HVOX fibroblasts and rat VFFbs. HVOX fibroblasts and rat VFFbs were treated with or without 10 ng/mL TGF-β1 and 3 µM verteporfin (VP) for 24 h. Expression of *CCN2* (**a**), *EDN1* (**b**), *FSTL1* (**c**), *ACTA2* (**d**), *COL1A1* (**e**), *FN1* (**f**), and *SERPINE1* (**g**) in HVOX and *Ccn2* (**h**), *Edn1* (**i**), *Fstl1* (**j**), *Acta2* (**k**), *Col1a1* (**l**), *Fn1* (**m**), and *Serpine1* (**n**) in rat VFFbs were analyzed by qPCR (data shown as mean ± SD; n = 3; *p < 0.05, control vs VP, TGF-β1 vs TGF-β1 + VP, Student’s *t*-test). Expression of αSMA (red) and collagen proteins (green) was evaluated by immunostaining (o; bar = 100 µm) and Western blotting (p; representative grouping of gels cropped from different parts of the same gel or from different gels; whole gels available in supplemental material). Cell nuclei counterstained with DAPI (blue; o).

**Figure 5:**
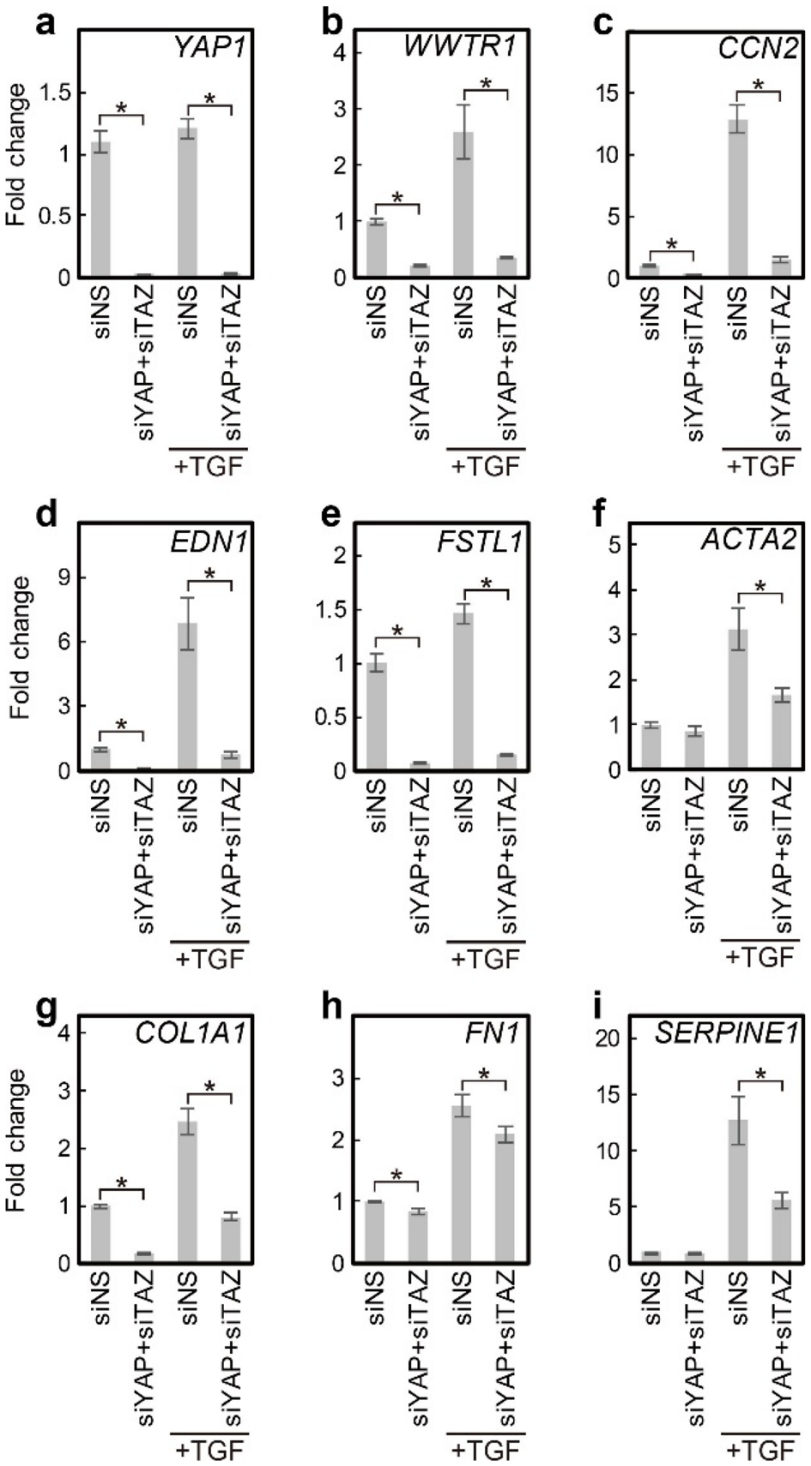
YAP and TAZ knockdown inhibited TGF-β1-induced fibroplastic phenotype changes in HVOX fibroblasts. HVOX fibroblasts transfected with siNS or both siYAP and siTAZ were treated with or without 10 ng/mL TGF-β1 for 24 h. Expression of *YAP1 *(**a**), WWTR1 (**b**), CCN2 (**c**), EDN1 (**d**), FSTL1 (**e**), ACTA2 (**f**), COL1A1 (**g**), FN1 (**h**), and SERPINE*1* (**i**) were analyzed by qPCR (data shown as mean ± SD; n = 3; *p < 0.05, siNS + TGF-β1 vs siYAP + siTAZ + TGF-β1, Student’s *t*-test).

The effects of verteporfin on YAP/TAZ activity and fibroplastic gene expression were further examined in HVOX fibroblasts. In addition, parallel experiments were completed in fibroblasts (rVFFbs) isolated and expanded from rat VFs to confirm interspecies consistency. In both HVOX and rVFFbs, 3 µM verteporfin significantly inhibited TGF-β1-induced *CCN2*, *EDN1*, and *FSTL1* (*Ccn2*, *Edn1*, and *Fstl1* in rat) expression (Fig. 4a–n). Vertporfin also reduced Type I collagen-α1 chain (*COL1A1*, *Col1a1*) and fibronectin (*FN1*, *Fn1*), fibrillary ECM components that accumulate in fibrotic tissues. Additionally, verteporfin decreased plasminogen activator inhibitor-1 (*SERPINE1*, *Serpine1*), a R-SMAD target gene^49^, as well as αSMA. Moreover, immunofluorescence staining and Western blotting confirmed verteporfin inhibited increased αSMA and type I collagen in TGF-β1 treated HVOX fibroblasts and rVFFbs (Fig. 4o and p). In addition, knockdown of YAP and TAZ via siRNA prevented TGF-β1-induced upregulation of *CCN2*, *EDN1*, *FSTL1*, *ACTA2*, *COL1A1*, *FN1*, and *SERPINE1* in HVOX fibroblasts (Fig. 5a–i). Cumulatively, these data indicate that YAP/TAZ activation is required for the TGF- β1-mediated fibroplastic phenotype in VFFbs.

**Figure 6:**
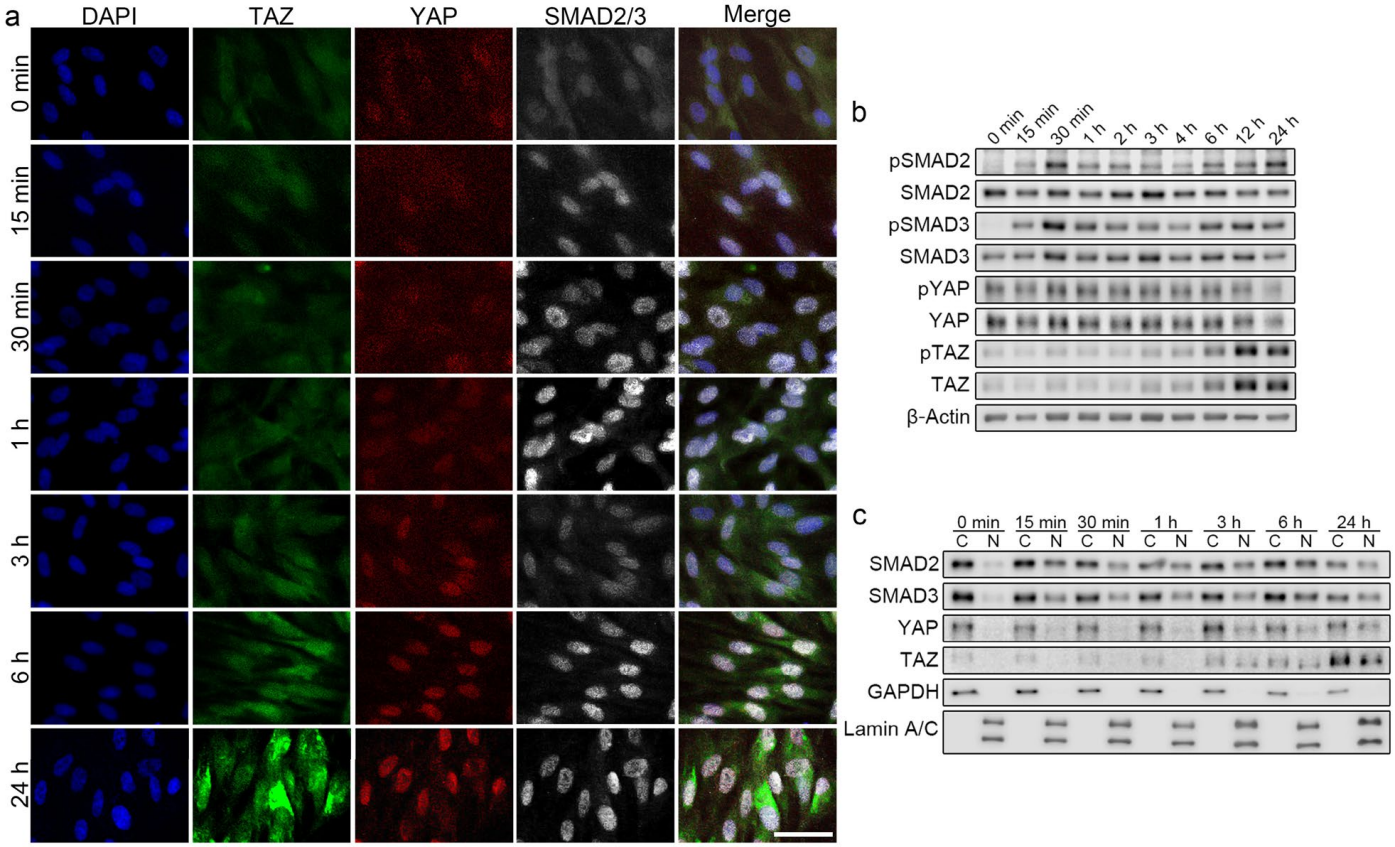
TGF-β1 concurrently activated SMAD2/3 and YAP/TAZ in HVOX fibroblasts. HVOX fibroblasts were treated with 10 ng/mL TGF-β1 for 0, 15, and 30 min and 1, 2, 3, 4, 6, 12, and 24 h (**a**,**b**). Distributional changes of YAP, TAZ, and SMAD2/3 were assessed by immunostaining (**a**; bar = 50 µm). Western blotting was performed to detect Ser465/467-phosphorylated SMAD2 (pSMAD2), SMAD2, Ser423/425-phosphorylated SMAD3 (pSMAD3), SMAD3, Ser127-phosphorylated YAP (pYAP), YAP, Ser89-phosphorylated TAZ (pTAZ), TAZ, and β-actin in the total cell fraction (**b**). HVOX fibroblast were treated with 10 ng/mL TGF-β1 for 0, 15, and 30 min and 1, 3, 6, and 24 h (**c**). Western blotting was performed to detect SMAD2, SMAD3, YAP, TAZ, GAPDH, and lamin A/C in the cytoplasmic (C) and nuclear (N) fractions. In (**b**) and (**c**), representative grouping of gels cropped from different parts of the same gel or from different gels; whole gels available in supplemental material.

TGF-β1 concurrently activated SMAD2/3 and YAP/TAZ. SMAD2/3 has a significant role in TGF-β1 pro-fibrotic signaling; we interrogated potential interactions between SMAD2/3 and YAP/TAZ. The time-course of SMAD2/3 and YAP/TAZ activation after TGF-β1 exposure was analyzed by immunofluorescence staining and Western blotting. SMAD2/3 nuclear localization and phosphorylation was observed in HVOX fibroblasts within 15 min of TGF-β1 treatment (Fig. 6a–c). Nuclear localization and phosphorylation of SMAD2/3 peaked at 0.5–1 h of TGF-β1 treatment, and this response was maintained to 24 h. Faint staining of YAP and TAZ in the nucleus was observed in untreated cells; this distribution was unchanged at 1 h of TGF-β1 treatment (Fig. 6a). Increased nuclear localization of YAP and TAZ was observed initially at 3 h and continued at 24 h. TAZ protein levels increased from 12 h, YAP seemed stable (Fig. 6b). Phosphorylation at Ser127 of YAP and Ser89 of TAZ was unchanged (Fig. 6b). Phosphorylation at these sites is necessary for cytoplasmic sequestration of YAP/TAZ through interactions with 14–3-3 protein, but not critical for inhibition of nuclear localization or binding to TEADs^50,51^. Consistent with immunofluorescence staining, YAP and TAZ increased in the nuclear fraction at 3, 6, and 24 h following TGF-β1 treatment (Fig. 6c). These data suggest that TGF-β/SMAD signaling activates YP/TAZ downstream of SMAD2/3 activation without modifying phosphorylation at Ser127 of YAP and Ser89 of TAZ in VFFbs.

### YAP and TAZ localized in close proximity to SMAD2/3 and supported SMAD2/3 nuclear accumulation

**Figure 7:**
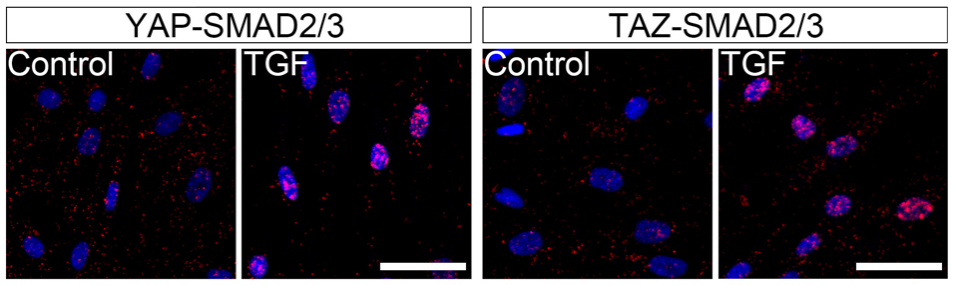
TGF-β1 stimulated localization of YAP/TAZ in close proximity to SMAD2/3 in HVOX fibroblasts. HVOX were treated with 10 ng/mL TGF-β1 for 24 h. Representative data of the proximity ligation assay using antibodies for YAP, TAZ and SMAD2/3 are shown (red = fluorescence raised by the assay, blue = cell nuclei stained with DAPI; bar = 50 µm).

**Figure 8:**
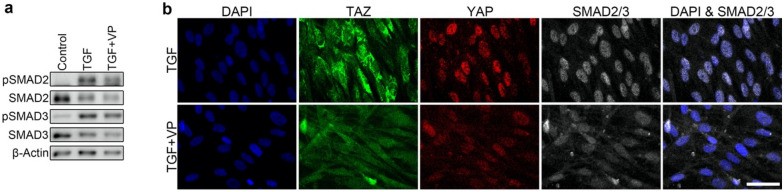
Verteporfin inhibited TGF-β1-induced SMAD2/3 nuclear accumulation without interfering with phosphorylation of SMAD2/3. HVOX fibroblasts were treated with or without 10 ng/mL TGF-β1 and 3 µM verteporfin (VP) for 24 h. Western blotting was performed to detect Ser465/467-phosphorylated SMAD2 (pSMAD2), SMAD2, Ser423/425-phosphorylated SMAD3 (pSMAD3), SMAD3, and β-actin in the total cell fraction (**a**; representative grouping of gels cropped from different parts of the same gel or from different gels; whole gels available in supplemental material). Nuclear localization of YAP/TAZ and SMAD2/3 were examined by immunostaining (**b**; bar = 100 µm).

TGF-β1 stimulated both YAP and TAZ to localize in close proximity to SMAD2/3 in the nuclei of HVOX fibroblasts at 12 h (Fig. 7). Verteporfin co-administered with TGF-β1 did not alter phosphorylation at Ser465/467 of SMAD2 and Ser423/425 of SMAD3 (Fig. 8a); however, SMAD2/3 nuclear localization was inhibited (Fig. 8b). Collectively, these data suggest YAP/TAZ binds to SMAD2/3 and supports SMAD2/3 nuclear accumulation rather than affecting phosphorylation of SMAD2/3 in VFFbs.

## Discussion

Vocal fold tissue health underlies our fundamental capacity for oral communication. However, the unique biophysical demands placed upon the tissue and their anatomic position increases the likelihood of iatrogenic injury, in addition to phonotrauma. Disordered voice is the most common communication disorder across the lifespan and is associated with staggering healthcare costs commensurate with conditions such as chronic obstructive pulmonary disease and asthma^3^. Inconsistent therapeutic strategies to address vocal fold injury and fibrosis prevail, potentially related to an incomplete understanding of relevant biochemical events in this highly specialized tissue. Our data suggest activation of YAP/TAZ, a core effector in the Hippo pathway, is required for TGF-β-induced SMAD2/3 activation and fibroplastic gene expression in human vocal fold fibroblasts in vitro and vocal fold fibrosis in vivo. These data are consistent with developmental and biochemical studies regarding significant roles for the Hippo pathway in pathological processes and its relevance to diverse signaling pathways^30^.

Iatrogenic injury dramatically increased expression and nuclear localization of YAP/TAZ within 3 days of injury. YAP/TAZ has been shown to mediate cell proliferation and survival; the rapid increase of YAP/TAZ activation after injury may be related to wound closure, as reported in other tissues^29,52^. More interestingly, YAP/TAZ nuclear localization persisted 14 and 90 days after injury, well into the ECM remodeling phase and maturation of fibrotic tissue^42^. These data, in addition to our in vitro data regarding a clear reduction of fibroplastic gene expression in response to YAP/TAZ inhibition, suggest YAP/TAZ may be an ideal target for treatment of VF fibrosis.

Because of the low affinity of SMADs to the genome at the SMAD-responsive element, SMADs require support from other transcriptional factors to support transcriptional activities^49^. Recent reports demonstrated physical interaction between YAP/TAZ-TEAD and SMADs, and *CCN2* is a common target of TEADs and SMAD2/3^53,54^. In addition, stabilization of nuclear SMAD2 by TAZ has been reported^41^. R-SMADs alone, regardless of phosphorylation, freely move across the nuclear envelope and require coupling with other molecules to accumulate in the nucleus^55^. Although activation of YAP/TAZ was delayed relative to SMAD2/3 in VFFbs in response to TGF-β1, YAP/TAZ, once activated, bound to SMAD2/3 and supported SMAD2/3 nuclear accumulation. Synergistic activation of SMAD2/3 and YAP/TAZ likely relies on the formation of a SMAD2/3, YAP/TAZ, and TEADs complex.

Phosphorylation at Ser127 of YAP and Ser89 of TAZ was not affected by TGF-β1. Activation of YAP/TAZ by TGF-β1 was, therefore, likely mediated by other factors. Since overexpression of YAP/TAZ can drive TEAD-dependent gene expression^56,57^, upregulation of TAZ induced by TGF-β1 could lead to activation of TAZ. Recent reports in TIG-1 and C3H10T1/2 fibroblasts also confirmed TGF-β1 increased TAZ protein, but not YAP, and suggested this finding was associated with a Smad3-independent, p38-mediated and redox-sensitive pathway^58,59^. However, the mechanism(s) of YAP/TAZ activation in TGF-β signaling cascade remain largely unknown and further investigation is required. Our in vivo data, however, suggest that YAP/TAZ are activated during fibroplastic tissue formation in the VF. YAP/TAZ and SMAD2/3 were synergistically activated by TGF-β1 in VF fibroblasts, and essential for TGF-β1-stiulated fibroplastic gene upregulation. These data, collectively, may implicate YAP/TAZ inhibition as an attractive therapeutic option for VF fibrosis.

## Methods

### Animals

All animal experiments were performed in accordance with relevant guidelines and regulations and approved by the Institutional Animal Care and Use Committee at the New York University Grossman School of Medicine. This manuscript follows the recommendations in the ARRIVE 2.0 guidelines. Ten, 14-week-old, female Sprague–Dawley rats were sacrificed for immunohistochemical analysis after vocal fold injury. One, 14-week-old, Sprague–Dawley rats was sacrificed for VFFbs isolation.

### Rat vocal fold injury

Unilateral VF injury was induced as previously described by our group and others^60^. Briefly, following paralytic anesthesia via intraperitoneal injection of ketamine hydrochloride (90 mg/kg) and xylazine hydrochloride (8 mg/kg), animals were placed on a custom operating platform in a near-vertical position, and the larynx was visualized via a 2.7-mm, 0° or 30° telescope (KarlStorz, Flanders, NJ) coupled to a camera and video monitor. The right VF was injured by separating the lamina propria from thyroarytenoid muscle by inserting a 25-gauge needle at the lateral edge of the right VF. The lamina propria was then removed with microforceps. Ten animals were randomized into six experimental groups based on time of sacrifice: 0 (e.g., uninjured control), 1, 3, 7, 14, and 90 days following injury. Following sacrifice, the larynx was harvested and prepared for immunohistochemistry.

### Human vocal fold fibroblast cell line

An immortalized human vocal fold fibroblast cell line, HVOX fibroblasts, created in our laboratory was employed for cell culture experimentation^43^. This cell line was treated with plasmocin (InvivoGen, San Diego, CA) for removal of mycoplasma and the lack of mycoplasma was confirmed via Venor™ GeM Mycoplasma Detection Kit (MilliporeSigma, St. Louis, MO). Cells in passages 11 to 20 were used. Cells were maintained in Dulbecco's Modified Eagle's Medium (DMEM) containing 10% fetal bovine serum (FBS) and 1% antibiotic/antimycotic (Life Technologies, Grand Island, NY) at 37 °C, 5% CO_2_. Following overnight serum starvation using FBS-free DMEM, cells were treated with 0.3–100 µM verteporfin (MilliporeSigma) and/or 10 ng/mL TGF-β1 (Life Technologies). Expression of YAP and TAZ was knocked down using the following siRNAs: human *YAP1*, ID: s20366 (siYAP); human *WWTR1*, ID: s20367 (siTAZ). The siRNAs were transfected using Lipofectamine RNAiMAX (Thermo Fisher Scientific) for 48 h before serum starvation.

### Rat vocal fold fibroblasts

The mucosa of the rat larynx was removed under a microscope and incubated in 500U/mL collagenase type II (MilliporeSigma) at 37 °C for 1 h. Cells were enzymatically released from the mucosa and undigested tissues were spread onto plastic dishes. The cells were expanded in DMEM containing 10% FBS, 5 ng/mL basic fibroblast growth factor (MilliporeSigma), and 1% antibiotic/antimycotic at 37 °C, 5% CO_2_. Cells in passage 5–10 were treated with 3 µM verteporfin (MilliporeSigma, St. Louis, MO) and/or 10 ng/mL TGF-β1.

### Quantitative real-time polymerase chain reaction (qPCR)

Cells were harvested at 24 h after treatment. Total RNA was extracted via the RNeasy Mini Kit (Qiagen, Valencia, CA) and reverse transcribed with a High-Capacity cDNA Reverse Transcription Kit (Applied Biosystems). The TaqMan Gene Expression kit (Life Technologies) and StepOne Plus (Applied Biosystems) were employed for quantitative analyses. Taqman primer probes for human *ACTA2* (Hs00426835_g1), *SERPINE1* (Hs00167155_m1), *COL1A1* (Hs00164004_m1), *FN1* (Hs01549976_m1), *CCN2* (Hs1026927_g1), *CYR61* (Hs00155479_m1), *EDN1* (Hs00174961_m1), *FSTL1* (Hs00907496_m1), *INHBA* (Hs01081598_m1), *YAP1* (Hs00902712_g1), *WWTR1* (Hs00210007_m1) and *GAPDH* (Hs02758991_g1), and rat *Acta2* (Rn01759928_g1), *Serpine1* (Rn01481341_m1), *Col1a1* (Rn01463848_m1), *Fn1* (Rn00569575_m1), *Ccn2* (Rn01537279_g1), *Edn1* (Rn00561129_m1), *Fstl1* (Rn00577634_m1) and *Gapdh* (Rn01462662_g1) were employed. The ΔΔCt method was employed with GAPDH as the housekeeping gene for quantification of relative expression.

### Immunohistochemistry and immunocytochemistry

Rat larynges were fixed and cryosections were prepared as described previously^60^. Cells were fixed using 4% paraformaldehyde. The sections and cells were permeabilized with 0.2% Triton-X, and subsequently incubated in phosphate buffered saline containing 0.05% Tween-20 and 1% bovine serum albumin. Primary and secondary antibodies are shown in Table S1; specimens were mounted via ProLong™ Gold Antifade Mountant with DAPI (Thermo Scientific, Waltham, MA). Images were captured using Zeiss Axio Observer, and LSM 700 confocal laser microscope (Carl Zeiss, Oberkochen, Germany).

### Cytotoxicity analysis

Media was obtained from cells cultured in 96-well plates with serum-free DMEM ± verteporfin for 24 h. LDH activity in the media was measured using CyQUANT LDH Cytotoxicity Assay kit (Thermo Fisher Scientific). Media was also obtained from cells killed with 1% TritonX-100 as a positive control. LDH activities were reported relative to the positive control and expressed as percentage. Live and dead cells were stained with calcein-AM and ethidium homodimer-1, respectively, according to the manufacturer’s protocol for the LIVE/DEAD Viability/Cytotoxicity Kit (Thermo Fisher Scientific). Cell images were captured using Zeiss Axio Observer. Live and dead cells in a 2.9mm^2^ region of interest (ROI) in the center of wells were counted; the percentage of dead cells relative to the total cells were reported.

### Western blotting

Following treatment, cells were washed with ice-cold PBS. Cells were then scraped and suspended in ice-cold PBS. Following snap centrifugation, cell precipitants were well-dissociated in lysis buffer: PBS supplemented with 0.05% IGEPAL CA-630 (MilliporeSigma), Halt Protease Inhibitor Cocktail (Thermo Scientific), Halt Phosphatase Inhibitor Cocktail (Thermo Scientific), 5 mM EDTA Solution, Calyculin A (Cell Signaling). The cell suspension was collected as a total cell fraction or centrifuged again. Supernatants were collected as a cytoplasmic fraction. The precipitated materials were washed once with the lysis buffer, suspended in the lysate buffer again, and used as a nuclear fraction. These fractions were combined with 4 × Laemmli Sample buffer (Bio-Rad) and 2-mercaptoethanol, and heated to 95 °C for 3 min. Samples were loaded on 8% sodium dodecyl sulfate–polyacrylamide gels and then transferred to PVDF membranes (Invitrogen) and blocked with 5% BSA (Fisher Scientific) for 90 min. Membranes were incubated with the primary and secondary antibodies shown in Table S1. Signals were detected using Odyssey Fc Imaging System (LI-COR Biosciences, Lincoln, NE) after incubation with SuperSignal™ West Dura Extended Duration Substrate (Pierce Biotechnology, Rockford, IL).

### Proximity ligation assay

Cells were fixed, permeabilized, and incubated with primary antibodies against YAP, TAZ, and SMAD2/3 as described in the immunocytochemistry methods above. Ligation with nucleotide chains and amplification of fluorescent probes were induced between YAP, TAZ, and SMAD2/3 in close proximity by using the Duolink PLA kit (MilliporeSigma). Signals were detected using LSM 700 confocal laser microscope.

### Statistical Considerations

 All in vitro experiments were repeated in triplicate, at least. Student’s *t*-test was performed to determine statistical significance between two groups. Cytotoxicity of verteporfin at different concentrations were compared from the control using Dunnet’s test followed by one-way analysis of variance test. *P* < 0.05 was determined as the threshold for significance.

## Supplementary Information

Supplementary Information.
