# Supplementary material for: Concurrent YAP/TAZ and SMAD signaling mediate vocal fold fibrosis
Source: Sci Rep. 2021 Jun 29;11:13484. doi: 10.1038/s41598-021-92871-z (PMC8241934; doi:10.1038/s41598-021-92871-z)
Supplement: Supplementary file 1 — Supplementary Information. [file 41598_2021_92871_MOESM1_ESM.pdf]

Table S1. Antibodies

Target protein	Provider	Product ID	Application	Host	Dilution	Conjugation
Primary antibody						
α-SMA	R&D Systems	ab21027	IHC	Goat	2 μg/mL	
α-SMA	MilliporeSigma	A2547	ICC<br>WB	Mouse	×1,000 (ICC)<br>×10,000 (WB)	
β-actin	Cell Signaling Technology	4970	WB	Rabbit	×10,000	
Collagen, type I	Abcam	ab34710	WB<br>ICC	Rabbit	×500 (ICC)<br>×1,000 (WB)	
GAPDH	Cell Signaling Technology	2118	WB	Rabbit	×10,000	
Lamin A/C	Santa Cruz Biotechnology	sc-376248	WB	Mouse	×10,000	
SMAD2	Cell Signaling Technology	5339	WB	Rabbit	×1,000	
SMAD2, Ser465/467-phosphorylated	Cell Signaling Technology	3108	WB	Rabbit	×500	
SMAD3	Cell Signaling Technology	9523	WB	Rabbit	×500	
SMAD3, Ser423/4235-phosphorylated	Abcam	ab52903	WB	Rabbit	×500	
SMAD2/3	R&D Systems	AF3797	ICC	Goat	×500	
TAZ	Atlas Antibodies	HPA007415	IHC, ICC	Rabbit	×200	
TAZ, Ser89-phosphorylated	Cell Signaling Technology	59971	WB	Rabbit	×500	
YAP/TAZ*	Cell Signaling Technology	8418	WB	Rabbit	×500	
YAP	Santa Cruz Biotechnology	sc-101199	ICC<br>WB	Mouse	×100 (ICC)<br>×1,000 (WB)	
YAP	Cell Signaling Technology	14074	IHC	Rabbit	×100	
YAP, Ser127-phosphorylated	Cell Signaling Technology	13008	WB	Rabbit	×500	
Secondary Antibody						
Goat IgG	Thermo Fisher Scientific	A21447	IHC, ICC	Donkey	×2,000	AlexaFluor647
Mouse IgG	Thermo Fisher Scientific	A31570	IHC, ICC	Donkey	×2,000	AlexaFluor555
Mouse IgG	Cell Signaling Technology	7076	WB	Horse	×20,000	HRP
Rabbit IgG	Thermo Fisher Scientific	A31572	IHC	Donkey	×2,000	AlexaFluor555
Rabbit IgG	Thermo Fisher Scientific	A32790	ICC	Donkey	×2,000	AlexaFluor488
Rabbit IgG	Cell Signaling Technology	7074	WB	Goat	×20,000	HRP

IHC: Immunohistochemistry. ICC: Immunocytochemistry. WB: Western blotting.

*, anti-YAP/TAZ antibody was used to detect TAZ in Western blotting.

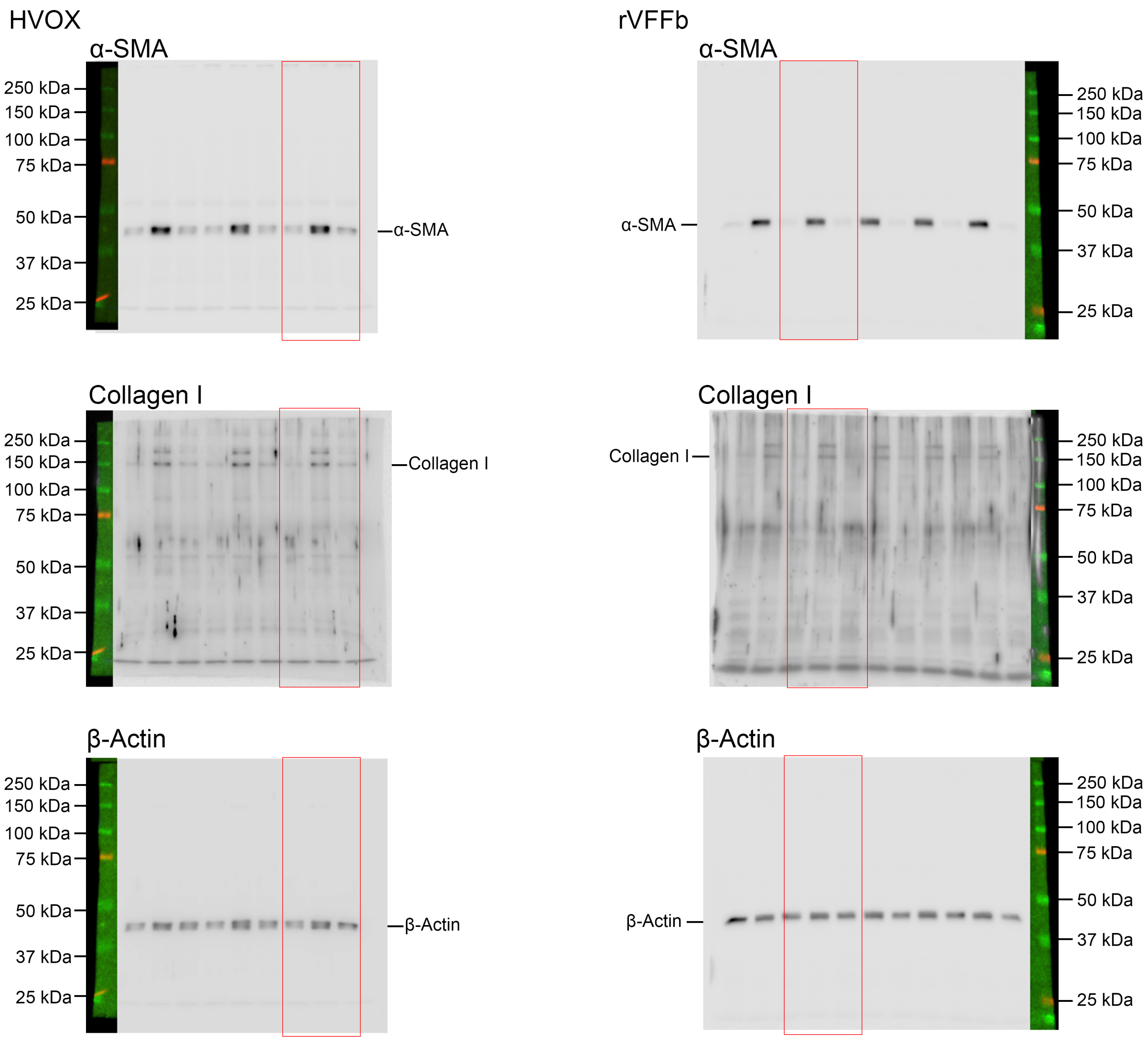

FIgure S1. Original images of Western blots in Figure 4p.

Blots denoted by the red rectanglars were cropped and are shown in the Figure 4p.

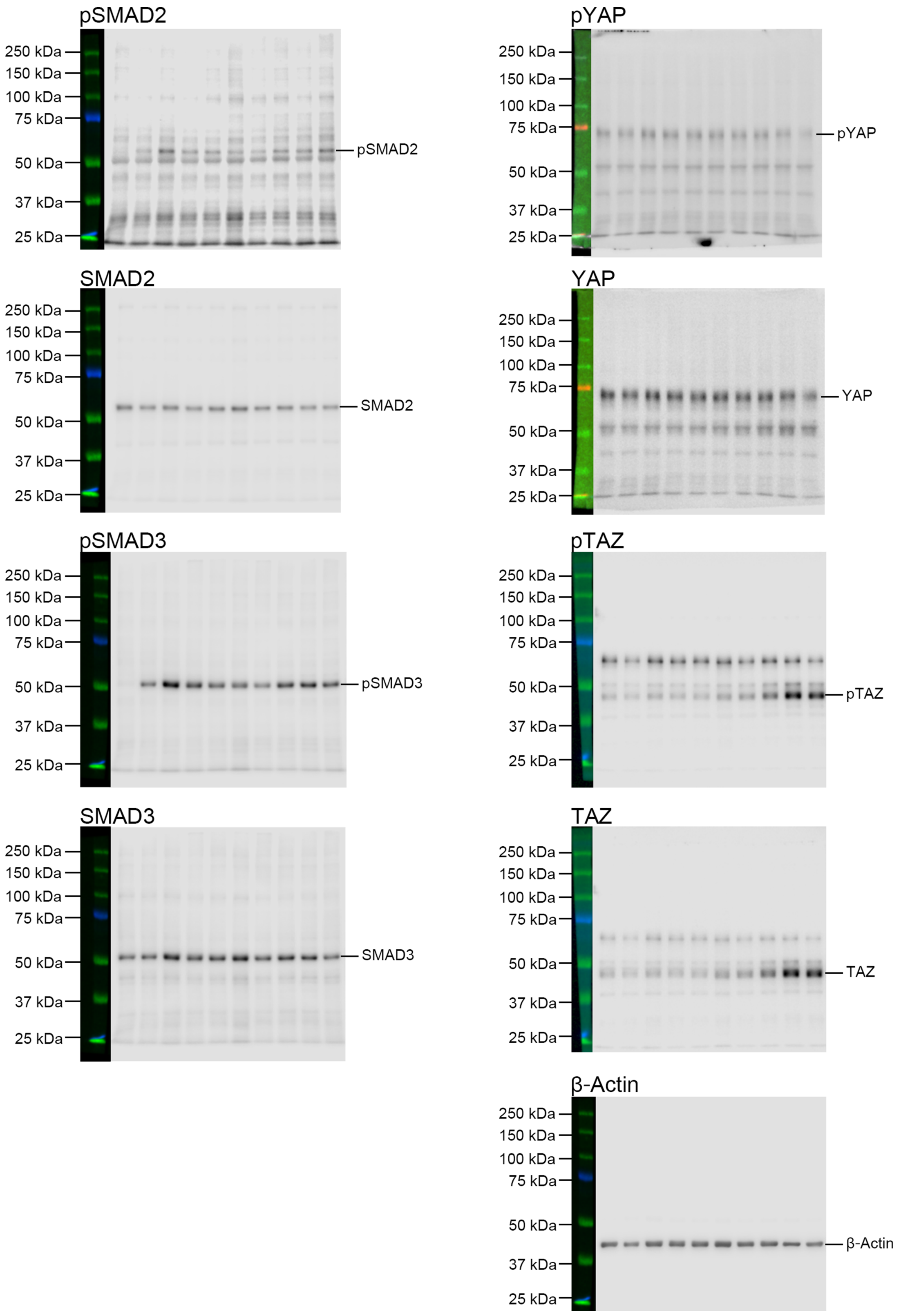

FIgure S2. Original images of Western blots in Figure 6b.

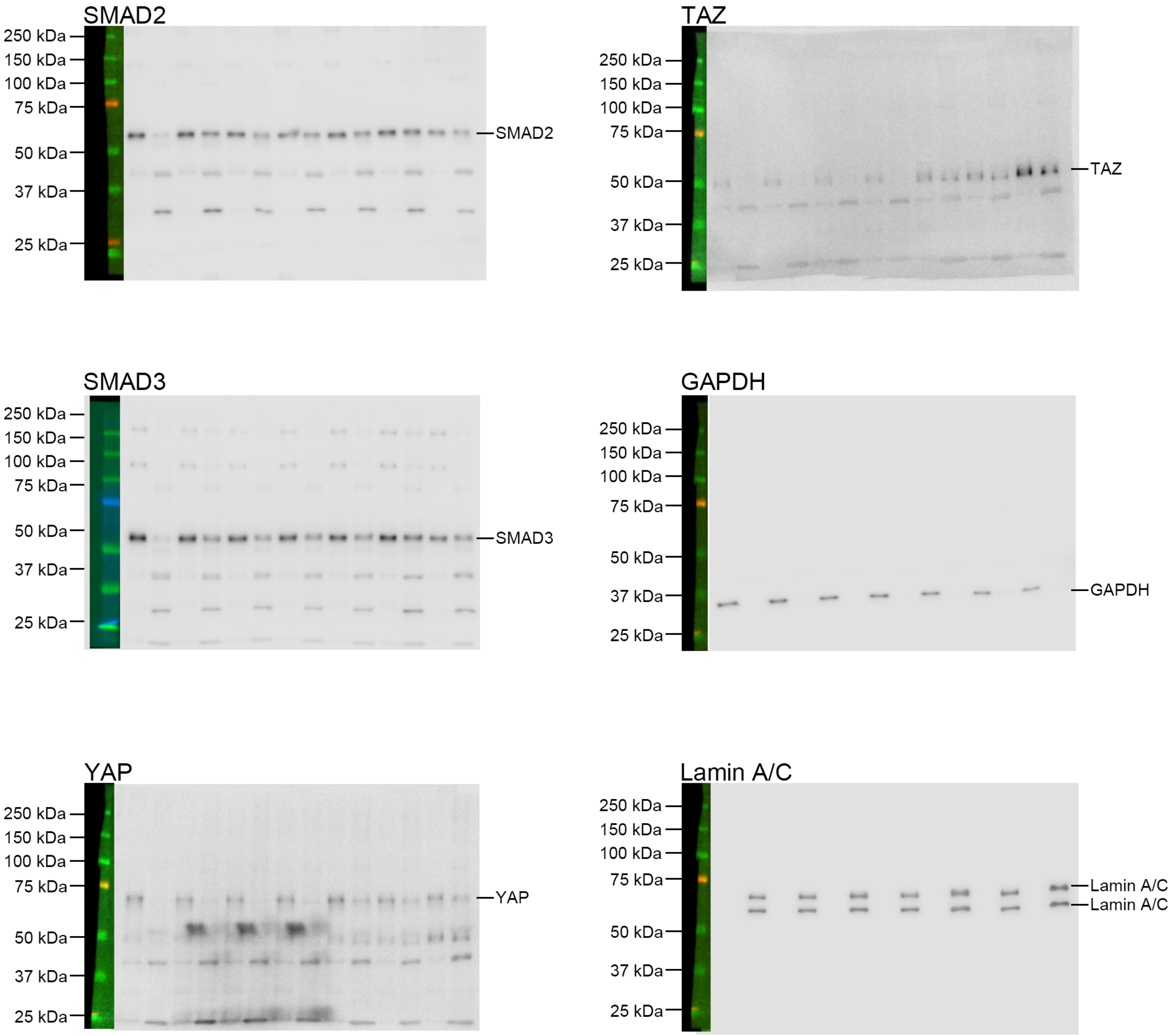

FIgure S3. Original images of Western blots in Figure 6c.

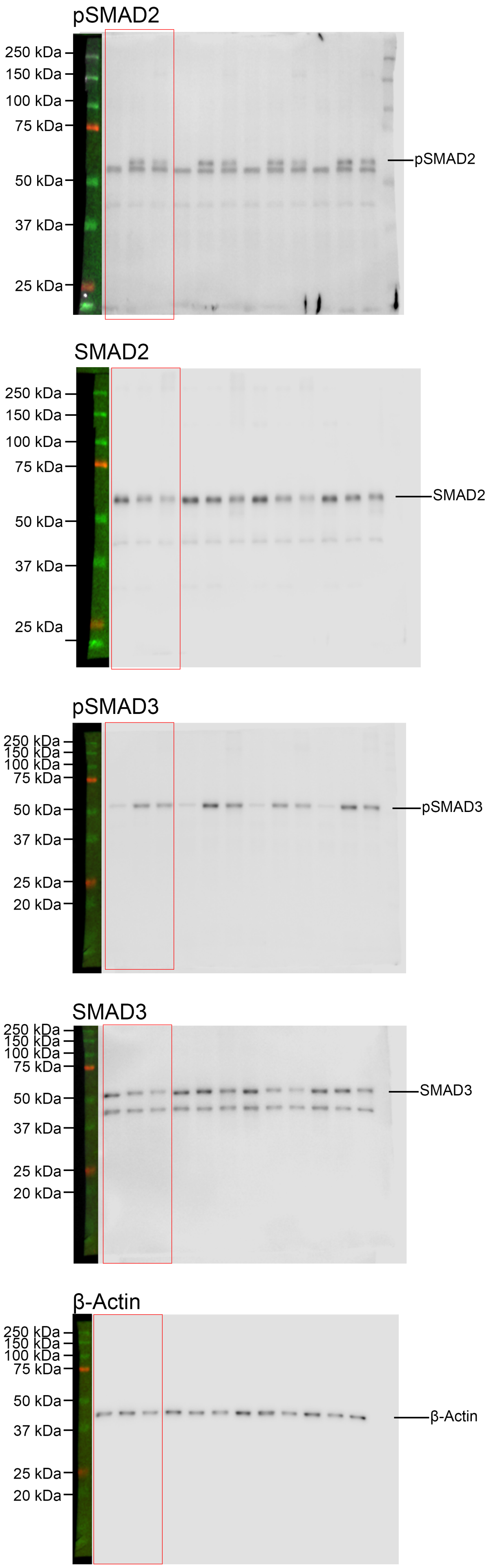

FIgure S4. Original images of Western blots in Figure 8a.

Blots denoted by the red rectanglars were cropped and are shown in the Figure 8a.
